# Risk factors for human brucellosis in agro-pastoralist communities of south western Uganda: a case–control study

**DOI:** 10.1186/s13104-015-1361-z

**Published:** 2015-09-04

**Authors:** Benon B. Asiimwe, Catherine Kansiime, Innocent B. Rwego

**Affiliations:** Department of Medical Microbiology, College of Health Sciences, Makerere University, P. O Box 7072, Kampala, Uganda; Department of Health Policy, Planning and Management, School of Public Health, College of Health Sciences, Makerere University, P. O Box 7072, Kampala, Uganda; Ecosystem Health Division, College of Veterinary Medicine, University of Minnesota, St. Paul, MN USA; Department of Biosecurity, Ecosystems and Veterinary Public Health, College of Veterinary Medicine, Animal Resources and Biosecurity, Makerere University, P. O Box 7062, Kampala, Uganda

**Keywords:** Brucellosis, Risk factors, Agro-pastoralists, Uganda

## Abstract

**Background:**

Brucellosis is a zoonosis of veterinary, public health and economic significance in most developing countries. The disease can result in permanent and disabling sequelae and considerable medical expenses in addition to loss of income due to loss of working hours. A case–control study was conducted in Nyabushozi, Kiruhura district, Uganda, so as to determine the risk factors for transmission of brucellosis to humans in these communities.

**Methods:**

We conducted a matched case–control study among participants in a previous study who were positive by the standard Serum Agglutination Test with titres ≥1:160. Controls were two neighbors for each case, matched by sex and age. A structured interviewer administered questionnaire was used to collect data on potential risk factors for brucellosis. Categorical variables were presented as proportions and their associations determined by Chi-square test. Bivariate analysis was performed to explore associations between the disease and the risk factors of brucellosis. Conditional logistic regression models were fitted to estimate independent associations between the disease and the risk factors using Odds Ratios and 95 % confidence intervals.

**Results:**

A total of 45 cases and 90 controls were interviewed. Of the 45 cases, 21 (46.7 %) were male while 44/90 (48.9 %) of the controls were female. The most significant risk factors for infection being an agro-pastoralist (*P* = 0.05), consumption of raw cow ghee (*P* = 0.03) and consumption of unpasteurized milk (*P* = 0.02).

**Conclusion:**

The greatest risk factors for acquiring brucellosis in the study area were being an agro-pastoralist, consumption of raw cow ghee and consumption of unboiled milk. We recommend dissemination of health education packages regarding risks and prevention measures for brucellosis in these communities.

**Electronic supplementary material:**

The online version of this article (doi:10.1186/s13104-015-1361-z) contains supplementary material, which is available to authorized users.

## Introduction

### Background

Brucellosis is the most common zoonosis accounting for more than 500,000 cases in the world annually [1]. Although human brucellosis is a notifiable disease in many countries official figures do not fully reflect the number of people infected each year [2]. The true incidence of brucellosis has been estimated to be between 10 and 25 times higher than what reported figures indicate [2]. The disease is caused by infection with bacteria of the genus *Brucella*, with four zoonotic species (genetically regarded as the variants of *Brucella melitensis* in genus *Brucella*): *B. abortus* is normally associated with cattle*, B. melitensis* with sheep and goats*, B. suis* with swine *and B. canis* with dogs [3]. The disease is often ignored in humans, and most cases go undiagnosed and untreated because of inaccurate diagnosis, and are thus treated as other diseases or as “fever of unknown origin”. Although any member of the public is at risk of getting brucellosis directly through contact with infected animal or material or indirectly through consumption of animal products, certain occupations such as veterinarians, butchers, abattoir workers, meat inspectors and farmers are known to be at a greater risk [4]. In some communities, consumption of home-made milk products is a risk factor for human brucellosis infections [5]. Additionally, other studies have found a statistically significant correlation between sero-positivity for brucellosis and age, sex, and the consumption of fresh cheese and cream made from unboiled milk [6].

In Uganda, previous surveys conducted revealed seroprevalence levels of 15.8 % within cattle herds in the pastoral dairy system in Mbarara district [7]; 12.6 % of informally marketed milk in urban Kampala city was contaminated with *B. abortus* at purchase, and the annual human incidence rate was estimated to be 5.8 per 10,000 people [8]. A recent sero-survey of brucellosis was carried out in the current study area and it was observed that up to 10 % of human participants in three sub-counties adjacent to Lake Mburo National Park in Kiruhura district were positive above the cut-off recommended by Ministry of Health [9]. This is poses a serious risk considering the close interaction between humans, domestic and wild animals, the high tourism activities in this locality, as well as the fact that the area is considered the milk basin of Uganda. The only studies to have been done in Uganda to determine the risk factors for human brucellosis [8, 10, 11] were carried out at the National Referral hospital and on participants living in urban Kampala, the capital city of Uganda. No study, to the best of our knowledge, has previously been carried out in rural agro-pastoralist communities where disease burden and risk factors are expected to be most high and easily amplified. This study, therefore, aimed at identifying risk factors for human brucellosis among agro-pastoral communities at this wildlife—domestic animals—human interface so as to inform public health.

## Methods

### Study area and population

**Fig. 1:**
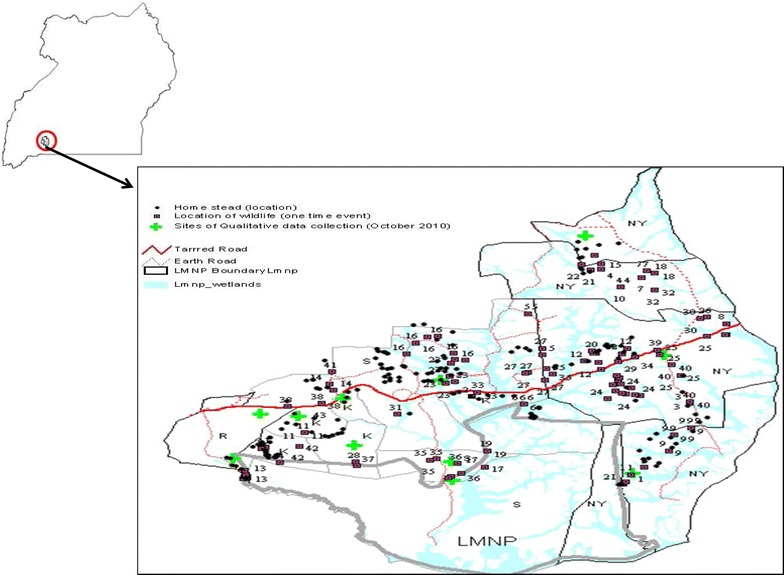
Map of Uganda showing the study area

The study was conducted in three sub-counties (Kanyaryeru, Nyakashashara and Sanga) of the South-Western rangelands of Nyabushozi county, Kiruhura district, Uganda. The sub-counties were selected because they are adjacent to Lake Mburo National Park (Fig. 1), and have domestic and wild-life grazing together, hence an added risk of disease transmission at this fragile interface. The surrounding areas are characterized by typical semi-arid savannah grasslands and support a variety of livelihoods: farmers, agro-pastoralists and semi-nomads. There is close interaction between wild and domestic animals and consequently humans, a key recipe for transmission of zoonotic diseases [12]. In 2011, a parallel study [13] recruited 300 households in the above three listed sub-counties for the purpose of assessing knowledge on zoonotic diseases focusing on brucellosis, in which a brucellosis sero-survey using the Serum Agglutination Test (SAT) was conducted [14]. The total number of participants was 576, of which 171 were from Kanyaryeru sub-county, 206 from Sanga sub-county and 199 from Nyakashashara sub-county.

### Study design

This was a matched case control study design conducted between June and December, 2013. A case was defined as an individual who tested positive by the standard Serum Aglutination Test (SAT), and because the area is considered endemic for the diseases, only titers ≥160 were considered as cases. Controls were two neighbors for each case, matched by sex and age, who had tested negative by the SAT at the same testing.

### Sample size and sampling procedures for the current study

In the sero-survey study described above, 29 of 171 participants from Kanyaryeru, 45 of 206 from Sanga and 41 of 199 from Nyakashashara were positive for brucellosis. As an endemic region, however, Ministry of Health guidelines stipulate that only titers ≥160 be considered as cases. By this guideline, in the current study, only 23 of 29 sero-positve individuals from Kanyaryeru; 17 of 45 from Sanga and 5 of 41 from Nyakashashara were considered cases therefore eligible for participation. All the 45 cases were recruited into the study. For each case, two age (±2 years) and sex matched controls were selected from households within the same neighborhood, and strictly in the same village. The sample size therefore was 45 cases and 90 controls, giving a total of 135 participants in the study.

### Data collection

All households in the sero-survey were listed and mapped by GIS in the previous study, making it easy to be revisited in the current study. A structured interviewer administered questionnaire was designed and pretested on five volunteers in the community. The questionnaire was translated from the original English version into the local language (Runyankole) and back translated to English by Makerere University Institute of Languages to ensure consistency and clarity in the community context. Data collected premised on potential risk factors for brucellosis, such as consumption of raw milk or other unprocessed dairy products, handling meat, and assistance of cows at delivery. During pre-testing of the study tool, additional information was gathered and some of the questions were modified to take care of the local cultural sensitivities.

### Data management and analysis

The outcome variables were presence or absence (0 for controls and 1 for cases) of brucellosis, while the predictor variables were types of livestock kept, handling of livestock and their products, consumption of animal products, history of brucellosis in the household, level of education, occupation, location of the household from the nearest Park boundary, and socio-demographic data such as age, sex and occupation. Data entry and coding were done in Epi-Data version 3.1 software and later exported to STATA version 12 for analysis. Categorical variables were presented as proportions and their association with other variables determined by the Chi-square test. Bivariate analysis was performed to explore associations between the disease and the risk factors of brucellosis. Conditional logistic regression models for matched case controls (1:2) were fitted to estimate independent associations between the disease and the risk factors. We used the variable id as a unique identifier to indicate the groups of matched case–control subjects. Inclusion of variables into the multivariable analysis was based on factors in bivariate analyses that either had *p* ≤ 0.2 and/or other variables known from literature to be associated with brucellosis. In all cases, a *p* value of ≤0.05 was considered as evidence of significant statistical association

### Ethical considerations

The study was approved by Makerere University School of Medicine Research and Ethics Committee and the Uganda National Council for Science and Technology. Informed written consent was obtained from all the participants; and only codes, not personal identifiers, were used on questionnaires for purposes of confidentiality. All cases had received medical treatment from the previous study (Additional file 1).

## Results and discussion

### Characteristics of cases and controls

A total of 45 cases and 90 controls were enrolled into the study. Of the 45 cases, 21 (46.7 %) were male while 44/90 (48.9 %) of the controls were female. Only 24 (53.3 %) of the cases and 37 (41.1 %) of the controls had attained primary education. Majority of the cases and controls were agro-pastoralists 68 (75.6 %) and 40 (88.9 %) respectively and most of the participants 33 (73.3 %) cases and 63 (70 %) controls mentioned prior knowledge of eating raw cow-ghee as a risk factor for brucellosis. Characteristics of the case and control subjects are shown in Table 1.

**Table 1:** Factors associated with brucellosis

Characteristics	Cases N = 45 (%)	Controls N = 90 (%)	Univariate analysis	*P* value
			Crude OR (95 %CI)	
Occupation				
Farmer	5 (11.1)	22 (24.4)	1	
Agro-pastoralist	40 (88.9)	68 (75.6)	3.62 (0.99–13.17)	0.05
Consumption of unboiled milk				
No	42 (93.3)	68 (75.6)	1	
Yes	3 (6.7)	22 (24.4)	4.53 (1.28–16.1)	0.02
Consumption of sour milk				
No	22 (48.9)	58 (64.4)	1	
Yes	23 (51.1)	32 (35.6)	0.56 (0.27–1.12)	0.10
Eat raw cow ghee				
No	6 (13.3)	32 (35.6)	1	
Yes	39 (86.7)	58 (64.4)	3.88 (1.41–10.7)	0.01
Rear goats and sheep				
No	26 (57.8)	42 (46.7)		
Yes	19 (42.2)	48 (53.3)	0.5 (0.20–1.25)	0.14
Knowledge of boiling milk as prevention				
No	9 (20)	34 (37.8)	2.5 (1.06–5.91)	0.04
Yes	36 (80)	56 (62.2)		
Frequency of wildlife on farms				
Never	7 (15.6)	25 (27.8)	1	
Rarely	14 (31.1)	29 (32.2)	0.6 (0.61–1.82)	0.33
Very often	24 (53.3)	36 (40)	1.27 (0.04–2.49)	0.04
Wildlife species grazing on farm				
Ungulate (buffalo, Impala)	36 (80)	59 (65.6)	1	
Non-ungulate (e.g. Zebra)	9 (20)	31 (34.4)	0.15 (0.20–1.05)	0.06

Level of significance ≤0.05

#### Bivariate analysis

All associations with a *p* ≤ 0.2 as well as all other variables known from literature to be associated with brucellosis were considered for multivariate analysis. Variables that were significantly associated with brucellosis at this stage of analysis were being an agro-pastoralists (OR = 3.62, CI 0.99–13.17), consumption of unboiled milk (OR = 4.53, CI 1.28–16.1), and eating raw cow-ghee (1.41–10.7) (OR = 3.88, CI 1.41–10.7). Details of the analysis are contained in Table 1.

### Conditional logistic regression

The most significant variables associated with brucellosis in the community were being an agro-pastoralist, consumption of unboiled milk and eating of raw cow ghee. Agro-pastoralists were 4.38 times more likely to suffer from brucellosis than crop farmers and participants who mentioned eating of raw cow ghee were 3.88 times more likely to suffer from the disease than those who did not. Consumption of unboiled milk was also a risk factor (P = 0.02) (Table 2).

**Table 2:** Bivariate (crude OR) and multivariate analysis (adjusted ORs) of the risk factors associated with brucellosis

Characteristics	Cases N = 45 (%)	Controls N = 90 (%)	Crude OR	Adjusted OR (95 %CI)	*P* value
Occupation					
Farmer	5 (11.1)	22 (24.4)	1		
Agro-pastoralist	40 (88.9)	68 (75.6)	3.62	4.38 (0.98–19.5)	0.05
Consumption of unboiled milk					
No	42 (93.3)	68 (75.6)	1		
Yes	3 (6.7)	22 (24.4)	4.53	0.11 (0.19–0.68)	0.02
Eat raw cow ghee					
No	6 (13.3)	32 (35.6)	1		
Yes	39 (86.7)	58 (64.4)	3.88	3.84 (1.12–13.13)	0.03
Rear goats and sheep					
No	26 (57.8)	42 (46.7)	1		
Yes	19 (42.2)	48 (53.3)	0.5	0.36 (0.11–1.14)	0.08

Level of significance ≤0.05

The present investigation determined risk factors for human brucellosis in agro-pastoralist communities using a matched case–control study. Studies in the East African region have reported occupation as a risk factor for acquiring brucellosis [15, 16], whereby animal handlers and Veterinarians are the most susceptible groups. There is need to promote health education about transmission, prevention and risk factors for brucellosis to the different occupations that handle animals and their products in order to reduce on the risk of acquiring the disease.

Consumption of unboiled milk was found to be a risk factor for brucellosis in our study. This is in agreement with other studies, for example in Pakistan, where individuals who consumed raw milk had higher odds of brucellosis seropositivity [17]. In addition, in Brazil the habitual intake of raw milk was found to be the probable cause of brucellosis [18]. A previous study in neighboring Kenya observed that un-pasteurized milk in addition to fermented milk were common vehicles for the transmission of brucellosis [15]. The current study area is considered the milk basin of Uganda, where milk from different farms/homesteads is pooled together at the trading centres for sale, posing a danger to the local tourists that are not necessarily in contact with animals. In fact, a recent case–control study in urban and peri-urban Kampala showed that living in urban areas was a risk factor for brucellosis [10], yet there is minimal contact with animas in these areas, supporting the argument that milk and related products bought in major dairy zones may be the source of disease. Public health programs should therefore focus on educating these communities about the risks of consuming unpasteurized milk, or dairy products made from unpasteurized milk, such as yoghurt and sour milk, which are a delicacy in the current study area (Fig. 1).

Consumption of raw cow ghee appeared to increase the risk of transmission (OR = 3.84, p = 0.03). This is contrary to a study done in Iran in which knowledge about the mode of brucellosis transmission by fresh cheese appeared to reduce the risk of acquiring the disease (OR = 0.44, *p* = 0.01) [5], and in Turkey where it was reported that consumption of mature cheese (more than 3 months) was found to reduce the risk of transmission of brucellosis [6]. However, since brucellosis usually occurs naturally in domestic animals and is transmitted to human beings by direct and indirect routes such as consumption of unpasteurized milk and dairy products [19, 20], there is need to sensitize communities on proper preparation and use of such milk products as cheese and ghee in rural settings.

In our study, having a member of the household who previously suffered from the diseases seemed to reduce risk of transmission of brucellosis (OR = 0.01, *p* < 0.001) in spite of the fact that the exposure of family members to the same epidemiological factors leads to more than one member of the family being infected. This was in contrast to findings in Iran where risk factors for infection were related to the existence of another case of brucellosis in the home (OR = 7.55, *p* = 0.0001) [5]. In our study, we hypothesized that preventive measures were taken after a family member was diagnosed with brucellosis so as to prevent further occurrence in the homestead. In this community, there is availability of educational materials on brucellosis that is provided through radio talk shows and the health centers in the area to anyone who has tested positive for, and is at risk of, brucellosis. We therefore recommend screening of all family members once a case is diagnosed in a homestead and health education packages for the surrounding communities spread more in the area. A limitation of this study is that the sample size was limited by the case definition of a titre of ≥1:160, leaving out most people who were exposed but still had lower titres.

### Conclusion

The greatest risk factors for acquiring brucellosis in the study area were being an agro-pastoralist, consumption of unboiled milk and eating raw cow ghee. We recommend the dissemination of health education packages emphasizing pasteurization of milk and consumption of processed milk products in these communities.

## Additional file

**Additional file 1.** Questionnaire: Risk Factors for Human Brucellosis in Agro-Pastoralist Communities of Nyabushozi County, South Western Uganda: A Case-Control Study.
