# Supplementary material for: Risk factors for human brucellosis in agro-pastoralist communities of south western Uganda: a case–control study
Source: BMC Res Notes. 2015 Sep 4;8:405. doi: 10.1186/s13104-015-1361-z (PMC4559326; doi:10.1186/s13104-015-1361-z)
Supplement: Supplementary file 1 — Additional file 1. Questionnaire: Risk Factors for Human Brucellosis in Agro-Pastoralist Communities of Nyabushozi County, South Western Uganda: A Case-Control Study. [file 13104_2015_1361_MOESM1_ESM.doc]

**Questionnaire: Risk Factors for Human Brucellosis in Agro-Pastoralist Communities of Nyabushozi County, South Western Uganda: A Case-Control Study**

**Part I: Socio- demographic characteristics of the respondents**

1. Code of the respondent_________________________________

Sub-county: **____________________________**Village name:**__________________** House No**:_____________**

1. Sex **:**

1= Male

2= Female

1. Age (Completed years): ____________
2. Religion:

1=Catholic

2= Anglican

3= Pentecostal

4=SDA

5=Moslem

6=other specify___________________

1. Marital status:

1= Married

2= co-habiting

3 = single (never married)

4=divorced

5=separated

6= widowed(er)

1. Completed educational status:

1= no formal education

2= primary

3 = secondary

4= tertiary

1. Occupation:

1= farmer

2=pastoralist

3= Agro- pastoralist

4=Semi-nomads

5=Butcher

6= Chef

7= other (specify) _______

### Part II: Questionnaire about risks for contracting brucellosis

1. Has any of your family members’ ever suffered from brucellosis?

1= Yes 2= No 3= do not remember

1. Does your occupation deal with animals?

1= Yes 2= No 3= do not remember

1. Do you consume unboiled milk?

1= Yes 2= No 3= do not remember

1. Do you consume sour milk?

1= Yes 2= No 3= do not remember

1. Do you eat raw ghee?

1= Yes 2= No 3= do not remember

1. Do you rear sheep and goats?

1= Yes 2= No 3= do not remember

1. Do you rear cows?

1= Yes 2= No 3= do not remember

1. Have you ever assisted in the delivery of cows or goats or sheep?

1= Yes 2= No 3= do not remember

1. Have you ever assisted in the abortion of any animal?

1 = Yes 2 = No 3 = do not remember

1. Do you know about cattle livestock vaccination against brucellosis?

1= Yes 2= No 3= do not know

1. Have you ever vaccinated your live stock against brucellosis?

1= Yes 2= No 3= do not remember

1. Do you know if unboiled milk can transmit brucellosis?

1= Yes 2= No 3= do not know

1. Do you know if pasteurized (boiled) milk can prevent transmission of brucellosis?

1= Yes 2= No 3= do not know

1. What is the distance, in Kilometers, from your home to the nearest boundary of the Lake Mburo national park?
2. How often does wildlife graze with your livestock on your farm?

1 = very often 2 = rarely 3 = never

1. List the wildlife species that graze with or near your livestock……………….. …………………………………………………………………………………..
